# The role of bone marrow stimulation in rotator cuff repair: a systematic review and meta-analysis

**DOI:** 10.1186/s40634-023-00589-w

**Published:** 2023-03-15

**Authors:** Guang Yang, Shangzhe Li, Chunyan Jiang, Hailong Zhang, Yi Lu

**Affiliations:** Sports Medicine Department, Beijing Jishuitan Hospital, No.31, Xin Jie Kou Dong Street, Xi Cheng District, Beijing, 100035 P. R. China

**Keywords:** Rotator cuff tear, Bone marrow stimulation, Meta-analysis

## Abstract

**Purpose:**

The objective of this study was to investigate whether RCR (rotator cuff repair) with BMS (bone marrow stimulation) can provide a lower retear rate and better shoulder function than arthroscopic RCR alone in rotator cuff tear (RCT) patients.

**Method:**

The PubMed, Cochrane Library, EMBASE and Web of Science databases were searched until Feb 2022. Risk of bias for randomized controlled trials was evaluated by two independent reviewers with Cochrane collaboration risk bias of tool, and that for cohort studies was evaluated with the Newcastle–Ottawa Scale (NOS). The primary outcome was the retear rate. Secondary outcomes included the American Shoulder and Elbow Surgeons (ASES) score, University of California, Los Angeles Shoulder Scale (UCLA) score, Constant-Murley score (CMS) and visual analogue scale (VAS) score. Subgroup analysis was performed to explore the effect of suture method and tear size on BMS procedure.

**Result:**

Five randomized controlled trials and four cohort studies with a total of 827 patients were included. The pooled retear rate between the RCR with BMS group and the RCR alone group was significantly different (17.5% vs. 28.9%; *P* < 0.0001). There were no differences in the ASES score, UCLA score and VAS score. The CMS was significantly higher in RCR with BMS group than the RCR alone groups (*P* = 0.02), while the difference was well below the MCID. RCR with BMS resulted in a significantly lower retear rate than RCR alone for large and massive RCTs (19.7% vs. 32.5%; *P* = 0.01).

**Conclusion:**

Compared with RCR alone, RCR with BMS can significantly reduce the retear rate in arthroscopic RCT patients while not clinically relevant differences were found. BMS may further reduce the retear rate of large and massive RCTs.

**Level of evidence:**

Level III; Systematic Review and Meta-analysis.

**Supplementary Information:**

The online version contains supplementary material available at 10.1186/s40634-023-00589-w.

## Introduction

Rotator cuff tear (RCT) is a common injury that causes shoulder pain and dysfunction. When conservative treatment fails, arthroscopic rotator cuff repair (RCR) can provide good clinical outcomes [1–4]. Despite advances in arthroscopic techniques, the retear rate of RCR is reported to range between 18% and 94%. Previous studies have shown that the retear rate of RCR is closely correlated with the initial biomechanical strength, tear size and tendon tissue quality [1, 5]. Therefore, providing a proper biological environment is one of the most effective ways to improve tendon-to-bone healing.

In 2009, Snyder et al. first used BMS combined with a single-row repair technique in RCT patients. By making several bone vents on the footprint area, a “crimson duvet”, which contains abundant marrow mesenchymal stem cells (MSCs), platelets and growth factors to promote tendon healing and reduce the retear rate after RCR, was formed [6]. While the effect of BMS remains controversial in the literature [7, 8]. Ruiz Ibán et al. reported no difference on constant score and EQ-5D-3 L in medium to massive RCT between groups [8]. Jo et al. reported no significant differences in clinical outcomes [7]. Sun et al. found that large-diameter microfractures may worsen rotator cuff healing in an animal study [9]. To our knowledge, there is no universally accepted conclusion on the effect of BMS on promoting the healing of tendons during RCR procedures.

The primary purpose of this study was to identify, summarize, and synthesize the evidence available of BMS in RCR and compare the retear rate between RCR combined with BMS and RCR alone in RCT patients. We hypothesized that in combination with RCR, BMS would significantly decrease retear rates and provide better clinical outcomes than RCR alone.

## Methods

### Search strategy

In accordance with the Preferred Reporting Items for Systematic Reviews and Meta-Analyses (PRISMA) guidelines, and this study was registered in PROSPERO (CRD42022351459), two independent reviewers performed this systematic review and meta-analysis. The PubMed, Cochrane Library, EMBASE and Web of Science databases were searched until Feb 2022. The following keywords were used: rotator cuff repair, rotator cuff tear, micro-fracture, bone marrow stimulation, multi-drilling, multiple channels, multiple drills, nano-fracture, bone marrow vents, multiple channeling, and marrow-stimulating. The more details were shown in the Appendix 1. The key words were restricted to the title or abstract. The references of all included studies were manually cross-referenced for further review to ensure a complete search of relevant studies not located in the original systematic search.

### Inclusion and exclusion criteria

Studies eligible for inclusion had to meet the following criteria: (1) two- or three-arm randomized controlled trials or cohort studies (Level of evidence: I-III) that reported clinical outcomes after primary arthroscopic RCR (ARCR), (2) BMS was used in one of the study groups, (3) at least 12 months of follow-up, and (4) patients older than 18 years. Exclusion criteria for the studies were as follows: (1) reviews and studies with no control group, (2) basic science studies such as cadaveric and animal studies, (3) studies investigating revision RCR, and (4) studies with incomplete data. Using these inclusion and exclusion criteria, the titles and abstracts of each of the papers were screened, and the full texts of potentially relevant studies were subsequently reviewed. A third senior author made a final decision on literature inclusion and exclusion if any discrepancy arose between the two independent reviewers.

### Quality assessment

The Cochrane collaboration risk-of-bias tool was used by two reviewers to evaluate the risk of bias for randomized controlled trials [10]. Any discrepancy was judged by another senior author to reach a consensus through discussion. With reference to the risk-of-bias tool items, we classified the study bias into high risk of bias, low risk of bias or unclear risk of bias. For cohort studies, the Newcastle–Ottawa Scale (NOS) was utilized to assess bias of the study. Studies with scores greater than or equal to 7 were classified as having a low risk of bias, those with scores of 4 to 6 were classified as having a moderate risk of bias, and those with scores less than 4 were classified as having a high risk of bias.

### Data extraction

All data from the included studies were extracted. The main data extracted in this study included author, publication year, research design, sample size, average age, sex, ARCR and BMS procedure. These data were imported into RevMan 5.3 meta-analysis software (The Cochrane Collaboration, Oxford, United Kingdom), data on the retear rate, the pre- and postoperative shoulder functional outcome scores, tear size and method of repair from all included studies were used for statistical analysis.

All patients were classified into two groups: the RCR with BMS group and the RCR alone group. The primary outcome of this study was the retear rate. Secondary shoulder functional outcomes consisted of the American Shoulder and Elbow Surgeons (ASES) score [11], University of California, Los Angeles Shoulder Scale (UCLA) score [12], Constant-Murley score (CMS) [13], and visual analogue scale (VAS) score [14, 15]. We compared the changes in the mean differences in the ASES score, UCLA score, CMS and VAS score to minimal clinically important difference (MCID) thresholds determined by previous rotator cuff studies: 17.9, 3, 6.7 and 1.4, respectively [16–18].

### Statistical analysis

For continuous outcomes, the mean difference was obtained and calculated from the inverse variance method. When the standard deviation was not provided for specific continuous outcomes, a well-established statistical formula described by Hozo et al. was used for imputation [19]. For dichotomous outcomes, the risk ratio (RR) was calculated using the Cochran-Mantel-Haenszel method. The heterogeneity of studies was tested by the standard χ^2^ test, and the I^2^ statistic was calculated to quantify heterogeneity. When the heterogeneity achieved significance (I^2^ < 50%), a fixed-effects model was used for the meta-analysis between two groups. When the heterogeneity did not achieve significance (I^2^ > 50%), a random-effects model was used for the meta-analysis between two groups. We calculated 95% confidence intervals (CIs) from all point estimates, and *p* < 0.05 was considered statistically significant for all outcome measurements.

## Results

### Literature search

**Fig. 1:**
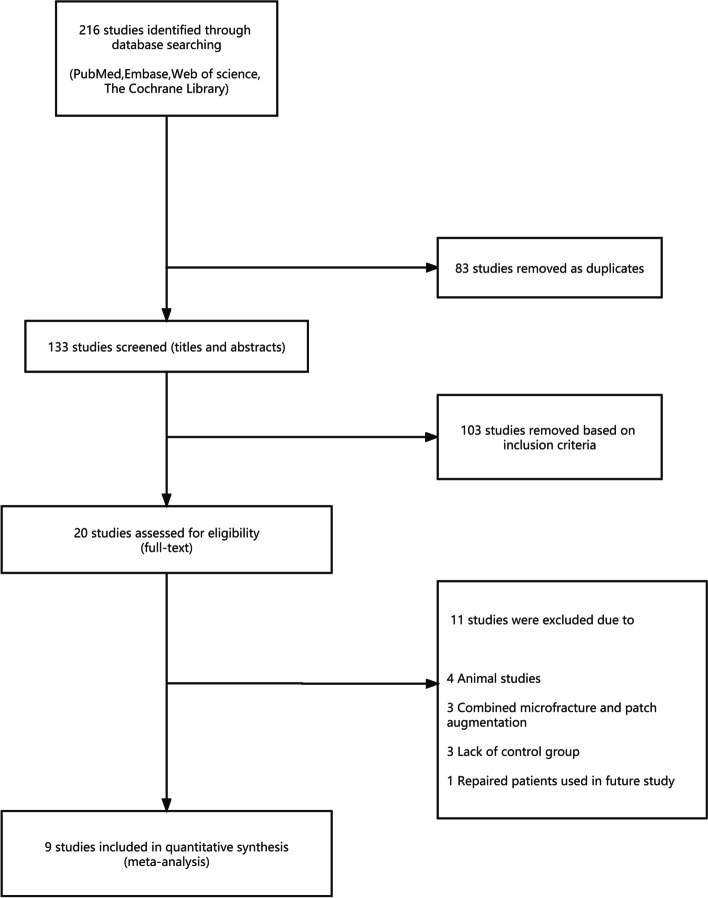
Search strategy results

The literature screen yielded 216 unique results. After screening the full texts, nine studies were deemed appropriate for inclusion in this study. There were five randomized controlled trials [8, 20–23], and four cohort studies [7, 24–26] (Fig. 1).

### Study quality assessment

**Fig. 2:**
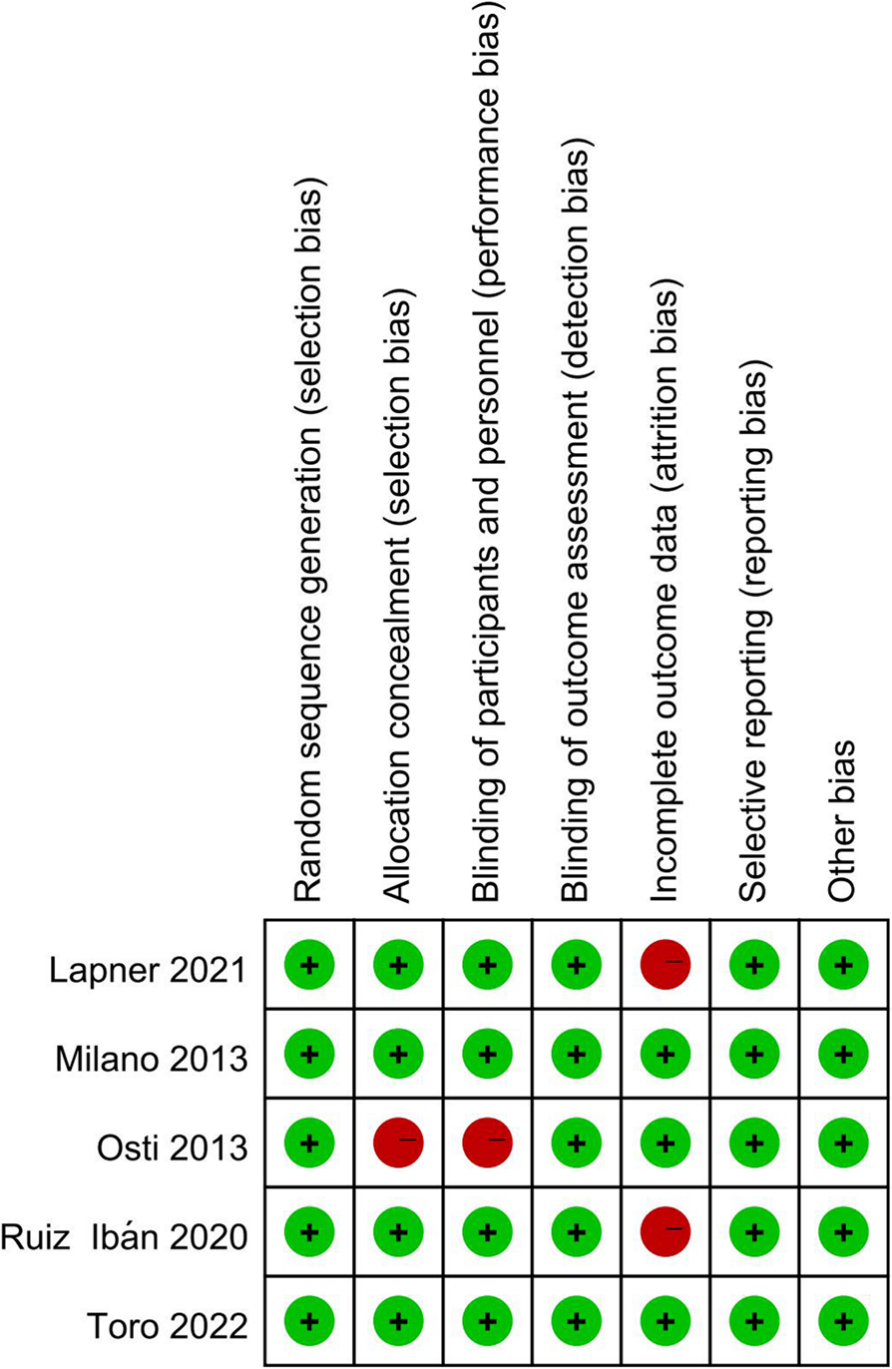
Detailed methodological assessment of the 5 included randomized controlled studies using the Cochrane risk of bias tool. Green, low risk of bias; red, high risk of bias; yellow, unclear or unknown risk of bias

In the quality evaluation of the five randomized controlled trials, allocation concealment was inadequate in one study by Osti et al., and it didn’t meet the rules of blinding as well because of inadequate patient blinding. Two studies did not meet our criterion for complete outcome data, one study by Lapner had a possibility of a type II error given the follow-up rate of 78%, while in another one by Ruiz Ibán, a power of only 0.5 was selected to limit the sample size. No study contained detection, selective reporting or other biases (Fig. 2).

In the quality evaluation of the four cohort studies, the NOS scores of two cohort studies were 9, and those of the other two studies were 7. Seven articles were of good quality, with a standardized research design and good research value (Table 1).

**Table 1:** Newcastle-Ottawa scale for risk of bias assessment of cohort studies included in the meta-analysis

Study	Selection				Comparability	Outcome			Overall
	Representativ-eness of Exposed Cohort	Selection of Nonexposed	Ascertainm-ent of Exposure	Outcome Not Present at Start		Assessment of Outcome	Adequate Follow- Up Length	Adequacy of Follow-Up	
Taniguchi et al. 2015 [26]	★	★	★	★	★★	☆	★	★	9
Jo et al. 2013 [7]	★	★	★	★	★	☆	★	★	7
Kim et al. 2020 [24]	★	★	★	★	★★	★★	★	☆	9
Pulatkan et al. 2020 [25]	★	★	★	★	★	★	★	☆	7

★,score of 1; ★★, score of 2; ☆,score of 0

### Patient demographics

There were a total of 827 patients included in the nine studies. Among the 827 patients, 749 had postoperative radiologic outcomes regarding the retear condition. The follow-up time ranged from 12 to 24 months. A total of 403 of 827 (48.7%) patients were in the RCR with BMS group, with an average age of 60.6 years (range, 58.1-64.7 years), 182 males and 221 females. Among them, 201 patients were treated by single-row repair, and 202 patients were treated by double-row repair (traditional double-row or suture-bridge technique). A total of 424 of 827 (51.3%) patients were in the RCR alone group, with an average age of 60.7 years (range, 57.8-64.3 years), 218 males and 206 females. Among them, 194 patients were treated by single-row repair, and 230 patients were treated by double-row repair (traditional double-row or suture-bridge technique). The author, publication year, research design, sample size, average age, sex, clinical outcome, method of BMS, and definition of retear are shown in Table 2. The method of suture repair, retear rates, functional outcome, VAS score, and duration of follow-up are shown in Table 3.

**Table 2:** Characteristics of the included studies

Author, year	Study	Sample size		Total	Gender(M/F)		Age, years		Bone marrow stimulation technique	Definition of retear	Clinical Outcomes	Bias grade	Follow-up
		BMS	Non-BMS		BMS	Non-BMS	BMS	Non-BMS					
Leonardo Osti 2013 [22]	RCT	28	29	57	16/12	14/15	61.2 (38-73)	59.8 (34-71)	• Used an awl for microfractures • 2-4 mm deep, 3-4 mm apart	Homogeneous low-intensity or partial high-intensity area (MRI)	UCLA,CMS	High risk	24 months
Giuseppe Milano 2013 [21]	RCT	35	38	73	22/13	19/19	60.6 ± 10.1	63.1 ± 9.2	• Used an awl for microfractures • 5 mm deep, 1.5 mm wide, 4 mm apart	Sugaya type III, IV, or V (MRI)	Dash,CMS	Low risk	24 months
Miguel Angel Ruiz Ibán 2021 [8]	RCT	36	33	69	14/22	18/15	60.1 ± 7.88	57.8 ± 10.7	• Used NanoFx Instrument for microfractures • 9 mm deep, 1 mm wide, 3-5 mm apart	Sugaya type IV, or V (MRI)	CMS,BPI,EQ-5D-3L	High risk	12 months
P. Lapner 2021 [20]	RCT	41	47	88	13/28	19/28	58.8 ± 8.8	59.5 ± 8.6	• Used a Kirschner wire for microfractures • 5 mm deep, 2 mm wide, 1 cm apart	Tendons were not in conti-nuity with an evidence of tearing (Ultrasound)	ASES,CMS,WORC	High risk	24 months
Felipe Toro 2022 [23]	RCT	62	61	123	29/33	35/26	58.9 ± 7.7	57.8 ± 9.2	“• Used an awl for microfractures • 3-5 mm deep, 3 mm apart”	Sugaya type IV, or V (MRI)	ASES,CMS	Low risk	12 months
Noboru Taniguchi 2015 [26]	CO	44	67	111	26/18	42/25	64.7 ± 1.4	64.3 ± 1.1	• Used a metal bar for microfractures • 3 mm wide, 3-5 mm apart	Sugaya type IV, or V (MRI)	JOA,UCLA	9	12 months
Chris Hyunchul Jo 2013 [7]	CO	57	67	124	25/32	33/34	58.89 ± 8.67	60.10 ± 7.94	• Used a rasp for multiple channeling • 10 mm deep, 2.1 mm wide, 4-5 mm apart	Sugaya type IV, or V (MRI)	ASES,CMS,UCLA,DASH,SST,SPADI	7	24 months
Chul Kim 2020 [24]	CO	56	42	98	26/30	23/19	64.6 ± 6.0	64.2 ± 5.5	• Used a custom-made awl for microfractures • 10 mm deep, 2 mm wide, 5 mm apart	French Society of Arthroscopy stage 3 or 4 (MRI and CTA)	ASES,UCLA,VAS,SSV	9	24 months
Anil Pulatkan 2020 [25]	CO	44	40	84	11/13	15/25	58.1 ± 9.7	59.2 ± 10.1	• Used a custom-made awl for microfractures • 5 mm deep, 1.3 mm wide, 4-5 mm apart	Sugaya type IV, or V (MRI)	CMS,VAS	7	24 months

*RCT* Randomized controlled study, *CO* Cohort study, *BMS* Bone marrow stimulation group, *Non-BMS* Standard surgery group, *ASES* American Shoulder and Elbow Surgeons’Form, *CMS* Constant-Murley outcome score, *UCLA* University of California, Los Angeles Shoulder Rating Scale, *VAS* visual analog scale, DASH-Hand Questionnaire, *JOA* Japanese Orthopaedic Association, *WORC* Western Ontario Rotator Cuff scores, *SST* Simple Shoulder Test, *SPADI* Shoulder Pain and Disability Index, *BPI* brief pain inventory

**Table 3:** Date of the included studies

Author	Method of repair	Retear rates		ASES		UCLA		Constant		VAS	
		BMS	Non-BMS	BMS	Non-BMS	BMS	Non-BMS	BMS	Non-BMS	BMS	Non-BMS
Lapner	double- row/suture bridge	4 of 36	4 of 39	84 ± 17	85 ± 12	–	–	83 ± 12	81 ± 16	–	–
Ruiz Ibán	double-row/suture bridge	7 of 36	14 of 33	–	–	–	–	84.8 ± 11.8	76.2 ± 24.1	0.9 ± 1.6	1.3 ± 2.1
Milano	single-row	12 of 35	18 of 38	–	–	–	–	94.5 ± 14	92.7 ± 16.7	–	–
Toro	single-row(79)/double-row(44)	3 of 48	7 of 47	96.9 ± 4.4	95.8 ± 7.6	–	–	92.9 ± 6	90.0 ± 11.2	–	–
Osti	single-row	2 of 28	3 of 29	–	–	32.6 ± 6	32.1 ± 5.8	92.3 ± 7.7	91 ± 7.3	–	–
Pulatkan	single-row	6 of 44	13 of 40	–	–	–	–	79.8 ± 8.4	75.5 ± 12.5	3.75 ± 1.75	3 ± 1.5
Jo	double-row(120)/single-row (control:4)	10 of 45	19 of 42	87.75 ± 17.05	88.14 ± 15.16	31.33 ± 4.36	30.99 ± 5.08	76.28 ± 13.53	75.61 ± 14.67	1.09 ± 1.66	0.99 ± 1.46
Taniguchi	double-row	4 of 44	16 of 67	–	–	–	–	–	–	–	–
Kim	single-row	17 of 56	15 of 42	87.4 ± 15.1	83.4 ± 13.5	28.5 ± 6.4	27.4 ± 6.7	–	–	1.2 ± 1.4	1.3 ± 1.4

All the date was extracted from involved study at final follow up

### Clinical outcomes

#### Retear rate

**Fig. 3:**
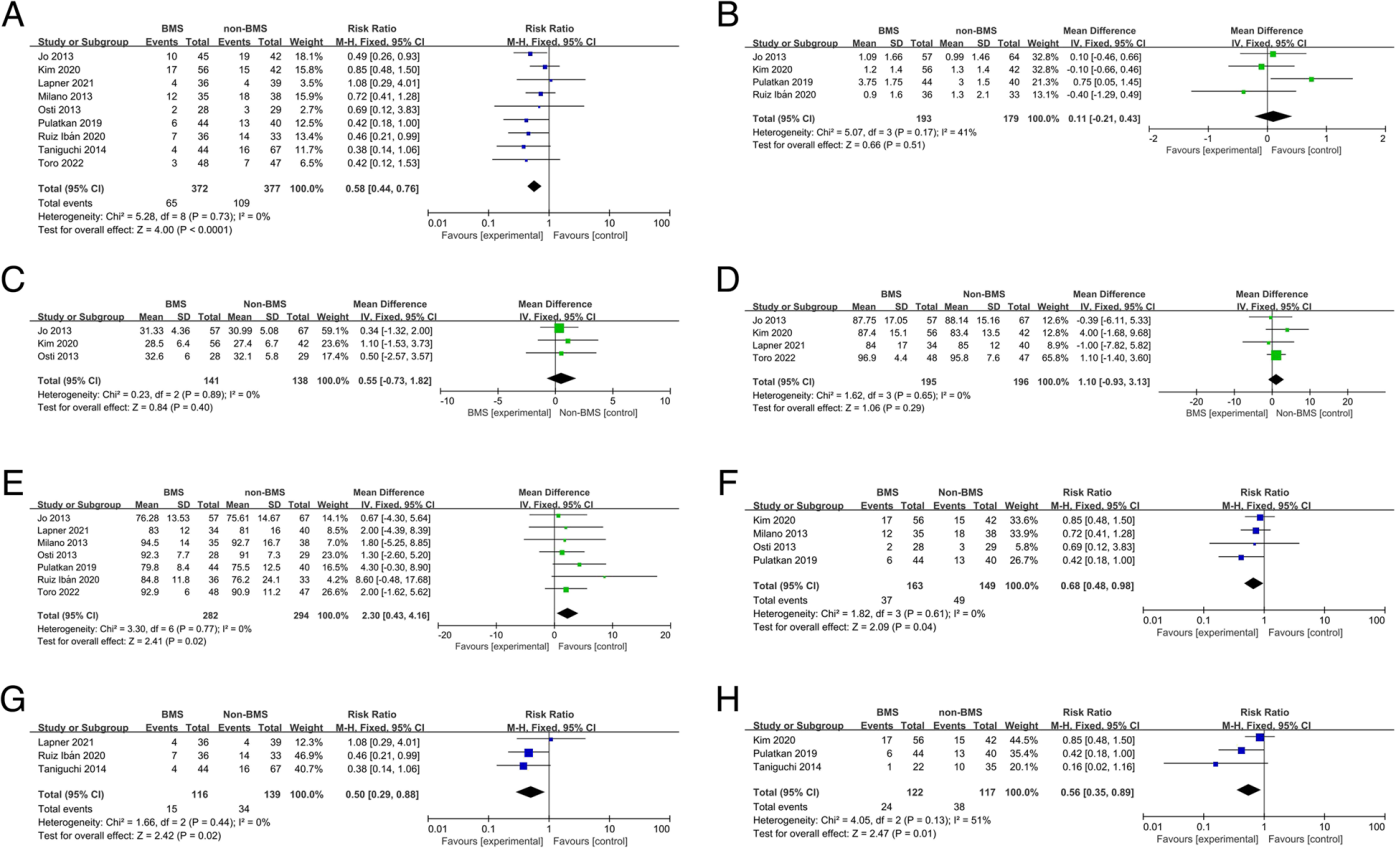
**A** Forest plot for the pooled retear rate. **B** Forest plot of the visual analogue scale score. **C** Forest plot of the University of California, Los Angeles Shoulder Scale score. **D** Forest plot of the American Shoulder and Elbow Surgeons score. **E** Forest plot of the Constant-Murley score. **F** Forest plot of the results of the subgroup analysis of single-row repair. **G** Forest plot of the results of the subgroup analysis of double-row repair. **H** Forest plot of the results of the subgroup analysis of large or massive tears. CI, confidence interval; IV, inverse variance; SD, standard deviation

The overall retear rate was 23.2% (174 of 749):17.5% (65 of 372) in the RCR with BMS group and 29.0% (109 of 377) in the RCR alone group. There was a significant difference between the two groups, with good homogeneity (I^2^ = 0%, *P* = 0.73; RR, 0.58; 95% CI, 0.44 to 0.76; *P* < 0.0001) (Fig. 3, A). When only pooling the retear rate of the five randomized controlled trials, which included 369 patients, there was a significant difference between the RCR with BMS group and the RCR alone group (15.3% vs. 24.7%; RR, 0.62; 95% CI, 0.42 to 0.93; I^2^ = 0%; *P* = 0.02). When only pooling the four cohort studies, which included 380 patients, there was also a significant difference in the retear rate between the RCR with BMS group and the RCR alone group (19.6% vs. 33.0%; RR, 0.55; 95% CI, 0.39 to 0.79; I^2^ = 0%; *P* = 0.001).

#### Pain

Four studies (372 patients) performed a VAS evaluation and revealed no significant difference between RCR with BMS and RCR alone (mean difference, 0.11; 95% CI, − 0.21 to 0.43; I2 = 41%; *P* = 0.51) (Fig. 3, B) [16, 20, 27, 28]. The mean difference in the VAS score did not reach the MCID threshold.

#### Functional outcomes

Three studies including 279 patients utilized the UCLA and found no significant difference between the two groups (mean difference, 0.55; 95% CI, − 0.73 to 1.82; I2 = 0%; *P* = 0.40) (Fig. 3, C) [7, 22, 24]. Four studies including 391 patients reported the ASES score at the final follow-up and found that it was not significantly different between the two groups (mean difference, 1.10; 95% CI, − 0.93 to 3.13; I2 = 0%; *P* = 0.29) (Fig. 3, D) [7, 20, 23, 24]. Seven studies including 576 patients used the CMS and found a significant difference between the two groups (mean difference, 2.30; 95% CI, 0.43 to 4.16; I2 = 0%; *P* = 0.02) (Fig. 3, E) [7, 8, 20–23, 25]. In each article that included the CMS, the BMS group had a higher CMS than the non-BMS group, but there were no significant differences.

#### Subgroup analysis

Two studies were excluded from the subgroup analysis because details on the suture repair method were lacking [7, 23]. Among nine studies, single-row repair was used in four (312 patients) [21, 22, 24, 25]. In the subgroup analysis on single-row repair, there was a significant difference in the retear rate between the RCR with BMS group and the RCR alone group (rate, 22.7% vs. 32.9%; RR, 0.68; 95% CI, 0.48 to 0.98; I^2^ = 0%; *P* = 0.04) (Fig. 3, F). Double-row/suture bridge repair was used in three studies [8, 20, 26], which included 255 patients. In the subgroup analysis on double-row/suture bridge repair, there was a significant difference in the retear rate between the RCR with BMS group and the RCR alone group (rate, 12.9% vs. 24.5%; RR, 0.50; 95% CI, 0.29 to 0.88; I^2^ = 0%; *P* = 0.02) (Fig. 3, G). The retear rate was lower in the double-row alone group than in the single-row alone group (24.5% vs. 32.9%). The retear rate was also lower in the double-row RCR with BMS group than in the single-row RCR with BMS group (12.9% vs. 22.7%). Although the suture repair method did not affect the tendency of BMS to reduce the retear rate, it was impossible to perform further statistical analysis because of the limited number of relevant studies.

Only three cohort studies involved large and massive RCTs [24–26]. There was a significant difference in the retear rate between the RCR with BMS group and the RCR alone group (rate, 19.7% vs. 32.5%; RR, 0.56; 95% CI, 0.35 to 0.89; I^2^ = 51%; *P* = 0.01) (Fig. 3, H). It was impossible to analyse other tear sizes because most of the studies did not mention the distribution and details of the sizes of the RCTs in their series.

## Discussion

The most important finding of the present study is that RCR combined with BMS can significantly reduce the retear rate in RCT patients. However, it cannot provide better clinical outcomes than RCR alone. BMS may further reduce the retear rate for large and massive RCTs, but its effect according to different suture methods for performing RCR has yet to be determined.

Snyder and Burns first proposed RCR with BMS using the term “crimson duvet” in 2009 [6], which describes a reddish purple-coloured clot formed from the bone marrow vent. It is known to contain MSCs, platelets with growth factors and vascular elements, which may provide important elements for tendon healing. In their subsequent study in 2020, 91% of their patients were satisfied with the results, 92% showed an intact rotator cuff at a minimum of 24 months of follow-up [29]. Pulatkan et al. and Yoon et al. both reported that the BMS group had a lower retear rate than the non-BMS group (33% vs. 14% and 46% vs. 19%, respectively) [31, 42]. In 2020, Ruiz Ibán reported lower retear in BMS group (19.4% vs 42.4%, *p* = 0.038) [8]. In 2019, Ajrawat et al. reported a statistically significant difference in pooled retear rates favouring BMS over RCR alone but included only two randomized studies and two cohort studies. When pooling the retear rate of the included randomized studies, there was no significant difference between the two groups [30]. In the present study, which incorporated a larger number of studies, the retear rate of the RCR with BMS group, regardless of the kind of study, was significantly lower than that of the RCR alone group (17.5% vs. 29.0%, *P* < 0.0001), which led us to conclude that the expected effect of BMS on reducing the retear rate in the RCR procedure was achieved.

In the present study, none of the shoulder functional outcomes in the RCR with BMS group except for the CMS were significantly different from those of the RCR alone group. This result is consistent with that of previous studies [31, 32]. The possible explanations are as follows: First, functional outcomes do not strongly correlate with retearing of the rotator cuff. Russell et al. reported no clinically significant differences in functional outcome scores or pain regardless of the structural integrity of the repair [33]. Second, the exudation of growth factors after BMS may result in a lack of long-term effects on tendon healing. Yoon et al. indicated that even though the “crimson duvet” covers the tendon-bone area, it probably subsequently vanishes into the subacromial space [34].

The size of RCTs may play a role in the efficiency of BMS. Taniguchi et al. reported no significant difference in the effect on medium RCTs between the two groups, while there were significant differences in the retear rate between the two groups for large to massive tears [26]. Milano et al. also performed a subgroup analysis for tear size, which suggested that the application of BMS could result in better healing for large tears [6]. This finding might indicate that the size of RCTs can affect the effect of BMS in reducing the retear rate. The present study showed a significant difference in the retear rate between the RCR with BMS group and the RCR alone group for large and massive tears (19.7% vs. 32.5%, *P* = 0.01). However, it should be noted that only three cohort studies were included in this subgroup analysis. Although BMS significantly reduced the retear rate in patients with large and massive RCTs, further investigation with more patient involvement and longer follow-up times is needed.

The suture method may also have an effect on the efficiency of BMS. Single-row and double-row repair techniques both achieve satisfactory clinical outcomes by restoring the footprint and providing adequate initial fixation [35–37]. In the present study, when subdivided into different repair method groups, the retear rate of the double-row RCR alone group was lower than that of the single-row RCR alone group (24.5% vs. 32.9%), which is comparable to the results reported in previous studies. Furthermore, the retear rate of the double-row repair RCR with BMS group was lower than that of the single-row repair RCR with BMS group (12.9% vs. 22.7%), which indicated that the additional BMS procedure did not change the effect of the repair technique on the retear rate of RCR. However, we were unable to obtain the *p* value for these analyses because of the limited number of relevant studies and the limitations of the relevant statistics.

## Limitation

Our study has several limitations, as follows. First, the present study only included nine studies involving 827 patients with 12-24 months of follow-up. Regarding the evaluations of retear RCRs and functions, the number of patients was still low, and long-term follow-up data are lacking. Second, there were only three cohort studies involved in the subgroup analysis of tear size and seven in the subgroup analysis of the RCR technique, which limits the generalizability of the conclusions of this study. Although the subgroup analysis reduced the heterogeneity, it would result in insufficient statistical power in the RCR with BMS group. Finally, only four studies were deemed to have a low risk of bias. This also reduces the credibility of the conclusion of this study.

## Conclusions

The present study demonstrated that RCR with BMS can significantly reduce the retear rate compared with RCR alone, especially in patients with large and massive RCTs. There were no significant differences in shoulder functional outcomes between the two groups except for the CMS, and all of the shoulder functional outcomes changes didn’t achieve the minimal clinically important difference (MCID). Neither single-row nor double-row RCR changed the tendency of BMS to reduce the retear rate.

## Supplementary Information

**Additional file 1.**
