# Supplementary material for: The role of bone marrow stimulation in rotator cuff repair: a systematic review and meta-analysis
Source: J Exp Orthop. 2023 Mar 15;10:27. doi: 10.1186/s40634-023-00589-w (PMC10014641; doi:10.1186/s40634-023-00589-w)
Supplement: Supplementary file 1 — Additional file 1. [file 40634_2023_589_MOESM1_ESM.docx]

Pubmed：

((((((((((((((((("Rotator Cuff Injuries"[Mesh]) OR (Cuff Injury, Rotator[Title/Abstract])) OR (Injury, Rotator Cuff[Title/Abstract])) OR (Rotator Cuff Injury[Title/Abstract])) OR (Rotator Cuff Tears[Title/Abstract])) OR (Rotator Cuff Tear[Title/Abstract])) OR (Tear, Rotator Cuff[Title/Abstract])) OR (Tears, Rotator Cuff[Title/Abstract])) OR (Rotator Cuff Tendinosis[Title/Abstract])) OR (Rotator Cuff Tendinoses[Title/Abstract])) OR (Tendinoses, Rotator Cuff[Title/Abstract])) OR (Tendinosis, Rotator Cuff[Title/Abstract])) OR (Rotator Cuff Tendinitis[Title/Abstract])) OR (Rotator Cuff Tendinitides[Title/Abstract])) OR (Tendinitis, Rotator Cuff[Title/Abstract]))) OR (Rotator Cuff Repair)) AND (((((((((micro-fracture) OR (bone marrow stimulation)) OR (multi-drilling)) OR (multiple channels)) OR (multiple drills)) OR (nano-fracture)) OR (bone marrow vents)) OR (multiple channeling)) OR (marrow-stimulating))

Embase：

#1 'rotator cuff rupture'/exp

#2 'rotator cuff repair':ab,ti

#3 'cuff injury, rotator':ab,ti OR 'injury, rotator cuff':ab,ti OR 'rotator cuff injury':ab,ti OR 'rotator cuff tears':ab,ti OR 'rotator cuff tear':ab,ti OR 'tear, rotator cuff':ab,ti OR 'tears, rotator cuff':ab,ti OR 'rotator cuff tendinosis':ab,ti OR 'rotator cuff tendinoses':ab,ti OR 'tendinoses, rotator cuff':ab,ti OR 'tendinosis, rotator cuff':ab,ti OR 'rotator cuff tendinitis':ab,ti OR 'rotator cuff tendinitides':ab,ti OR 'tendinitis, rotator cuff':ab,ti

#4 #1 OR #2 OR #3

#5 'micro fracture':ab,ti OR microfracture:ab,ti OR 'bone marrow stimulation':ab,ti OR 'multi drilling':ab,ti OR multidrilling:ab,ti OR 'multiple channels':ab,ti OR 'multiple drills':ab,ti OR 'nano fracture':ab,ti OR nanofracture:ab,ti OR 'bone marrow vents':ab,ti OR 'multiple channeling':ab,ti OR 'marrow stimulating':ab,ti

#6 #4 AND #5

Cochrane Library：

ID Search

#1 MeSH descriptor: [Rotator Cuff Injuries] explode all trees

#2 ("rotator-cuff repair"):ti,ab,kw

#3 #1 OR #2

#4 (micro-fracture):ti,ab,kw OR (microfracture):ti,ab,kw OR (bone marrow stimulation):ti,ab,kw OR (multi-drilling):ti,ab,kw OR (multidrilling):ti,ab,kw

#5 (multiple channels):ti,ab,kw OR (multiple drills):ti,ab,kw OR (nano-fracture):ti,ab,kw OR (nanofracture):ti,ab,kw OR (bone marrow vents):ti,ab,kw

#6 (multiple channeling):ti,ab,kw OR (marrow-stimulating):ti,ab,kw

#7 #4 OR #5 OR #6

#8 #3 AND #7

Web of Science：

#1 microfracture (Topic) or bone marrow stimulation (Topic) or multidrilling (Topic) or multiple channels (Topic) or multiple drills (Topic) or nanofracture (Topic) or footprint preparation (Topic) or bone marrow vents (Topic) or multiple channeling (Topic) or marrow-stimulating (Topic)

#2 rotator cuff repair (Topic) or rotator cuff tear (Topic) or rotator cuff injury (Topic) or rotator cuff tears (Topic)

#3 #1 and #2
